# Data detailing the platelet acetyl-lysine proteome

**DOI:** 10.1016/j.dib.2015.09.020

**Published:** 2015-10-09

**Authors:** Joseph E. Aslan, Larry L. David, Owen J.T. McCarty

**Affiliations:** a Departments of Biomedical Engineering, Portland, OR 97239, USA; b Cell and Developmental Biology, Portland, OR 97239, USA; c Knight Cardiovascular Institute, Portland, OR 97239, USA; d Division of Hematology & Medical Oncology School of Medicine, Portland, OR 97239, USA; e Proteomics Shared Resource, Oregon Health & Science University, Portland, OR 97239, USA

**Keywords:** Acetylation, Hemostasis, Platelets, Proteomics

## Abstract

Here we detail proteomics data that describe the acetyl-lysine proteome of blood platelets (Aslan et al., 2015 [1]). An affinity purification – mass spectrometry (AP-MS) approach was used to identify proteins modified by Nε-lysine acetylation in quiescent, washed human platelets. The data provide insights into potential regulatory mechanisms of platelet function mediated by protein lysine acetylation. Additionally, as platelets are anucleate and lack histone proteins, they offer a unique and valuable system to study the regulation of cytosolic proteins by lysine acetylation. The mass spectrometry proteomics data have been deposited to the ProteomeXchange Consortium (Vizcaino et al., 2014 [2]) via with PRIDE partner repository with the dataset identifier PXD002332.

**Specifications table**

**Table:** 

Subject area	Biology
More specific subject area	Proteomic analysis
Type of data	MS data
How data was acquired	Data acquired on a Thermo Orbitrap Fusion Tribrid Mass Spectrometer configured with an EasySpray NanoSource
Data format	.raw (raw mass spectrometry data files)
Experimental factors	Washed human platelet lysates digested with trypsin.
Experimental features	Acetyl-lysine peptides from platelet lysate digests were enriched using Cell Signaling PTMScan Acetyl-Lysine Motif Kit.
Data source location	Proteomics Shared Resource, Oregon Health & Science University, Medical Research Building Room 521, 3181 SW Sam Jackson Park Road, Portland, OR 97239 USA
Data accessibility	Deposited to the ProteomeXchange with identifier PXD002332. http://proteomecentral.proteomexchange.org/cgi/GetDataset?ID=PXD002332

Value of the data

• This data provides the first description of the platelet acetyl-lysine proteome.
• Platelets – the cellular mediators of hemostasis and thrombosis – are anucleate and lack histones, yet they are molecularly equipped to regulate and execute adhesion, cytoskeletal remodeling, metabolic, secretion, apoptotic and other processes common to cellular function and physiology. Accordingly, platelets serve as an ideal cellular model to study roles of lysine acetylation in cell biology apart from roles in transcriptional regulation.
• The data suggest roles for lysine acetylation in the regulation of actin cytoskeletal dynamics, mitochondrial metabolic processes and other cellular activities in platelets.
• Given the developing roles of lysine acetylation in the regulation of diverse cellular activities, this data is important for understanding the molecular basis of platelet function.
• As aspirin – a prevalent antiplatelet drug – acts as a generalized acetylation agent that is capable of modifying lysine residues by Nε-acetylation, the data offers insights into potential secondary targets of aspirin that may impact cellular function.

## 1 Data, experimental design, materials and methods

### 1.1 Data

The data in the PRIDE Archive provide the first description of the acetyl-lysine proteome of platelets – the cellular mediators of hemostasis and thrombosis [3].

### 1.2 Preparation of washed human platelets

Purified platelets were isolated from 100 ml human venous blood drawn from healthy volunteers by venipuncture into sodium citrate in accordance with an IRB-approved protocol at Oregon Health & Science University as previously described [1], [4]. Briefly, citrated blood was centrifuged at 200*g* (20 min) to obtain platelet rich plasma (PRP). PRP was centrifuged at 1000*g* (10 min) in the presence of prostacyclin (0.1 μg/ml). PRP-isolated platelets were resuspended in modified HEPES/Tyrode buffer (129 mM NaCl, 0.34 mM Na_2_HPO_4_, 2.9 mM KCl, 12 mM NaHCO_3_, 20 mM HEPES, 5 mM glucose, 1 mM MgCl_2_; pH 7.3) and washed one time with centrifugation at 1000*g* for 10 min in modified HEPES/Tyrode buffer. Purified platelets (>97.5% purity as determined by flow cytometry) were collected into in modified HEPES/Tyrode buffer for proteomics analyses described below.

### 1.3 Mass spectrometry and proteomics analyses

#### 1.3.1 Sample preparation and digestion

Washed human platelets were spun at 1000*g* for 10 min. The resulting pellet was suspended in 5 ml of deionized 8 M urea containing 10 mM triethylammonium bicarbonate (TEAB) supplemented with deacetylase and sirtuin inhibitors (10 mM nicotinamide and 10 μM SAHA). The platelet samples were then lysed by probe sonication (Model 60 Sonic Dismembrator, Fisher Scientific) at a 15 W setting for 15 s, with cooling on ice for 30 s for 3 treatments. Proteins were reduced with 18 μl of 1.25 M dithiothreitol and incubation at 55 °C for 30 min. Samples were then cooled to room temperature and cysteines were alkylated with the addition of 500 μl of 100 mM idoacetamide (15 min incubation). Sample was then diluted with 15 ml of 10 mM TEAB (+10 mM nicotinamide and 10 μM SAHA) and digested overnight at 37 °C with slow rotation following addition of 200 μg of MS Grade Trypsin (Thermo Scientific) dissolved in 200 μ1 of 1 mM HCl. Following digestion, the sample was acidified with TFA at a final 1% concentration and centrifuged at 2000*g* for 15 min. Next, peptides in the supernatant were solid phase extracted using a Sep-Pak Plus C18 cartridge (waters) and eluted in 5 ml of 40% acetonitrile, 0.1% TFA. The final recovered peptides had a mass of 2.5 mg, as determined by BCA assay using BSA as a standard (Thermo Scientific). The peptides were then shell frozen using a dry ice bath and dried in a vacuum centrifuge for 20 h without heating.

#### 1.3.2 Acetyl-lysine peptide enrichment

Acetylated peptides were purified using a PTMScan Acetyl-Lysine Motif Kit (Cell Signaling) using 80 μl of immunoaffinity beads following the manufacturer׳s recommendations. Affinity purified peptides were then solid phase extracted using a Sep-Pak light C18 cartridge and eluted in 1 ml of 40% acetonitrile, 0.1% TFA. Following vacuum centrifugation, the peptides were dissolved in 20 μl of 10 mM ammonium formate (pH 10) and separated by 2-D chromatography using tandem reverse phase separations at high and low pH. A Dionex NCS-3500RS UltiMate RSLCnano UPLC system was used for sample loading and 2nd dimension reverse phase separation, and a Dionex NCP-3200RS UltiMate RSLCnano UPLC system was used for dilution of the 1st dimension reverse phase eluent. The sample was injected for 10 min onto a NanoEase 5 μm XBridge BEH130 C18 300 μm×5 cm column (Waters) at 3 μl/min in a mobile phase containing 10 mM ammonium formate (pH 10), 2% acetonitrile (ACN). Next, peptides were eluted by sequential injection of 20 μl volumes of 20, 24, 28, and 40% ACN in 10 mM ammonium formate (pH 10) at a 3 ul/min flow rate. Eluted peptides were then diluted at a tee with mobile phase containing 0.1% formic acid at a 12 ul/min flow rate and bound to an Acclaim PepMap 100 μm×2 cm NanoViper C18, 5 μm trap column on a switching valve. Following loading, the trap column was switched on-line to a PepMap RSLC C18, 2 μm, 75 μm×25 cm EasySpray column (Thermo Scientific). Peptides were then separated at low pH in the 2nd dimension using a 7.5–30% ACN gradient over 83 min in mobile phase containing 0.1% formic acid at a 300 nl/min flow rate.

#### 1.3.3 Mass spectrometry data acquisition

Tandem mass spectrometry data was collected using a Thermo Orbitrap Fusion Tribrid Mass Spectrometer configured with an EasySpray NanoSource (Thermo Scientific). Survey scans were performed in the Orbitrap mass analyzer, and data-dependent MS2 scans in the linear ion trap following HCD fragmentation in the instrument׳s ion routing multipole, as detailed [1]. Peptides were identified using Protein Discoverer, version 1.4 (Thermo Scientific), using Sequest HT, a human Swiss-Prot database downloaded in April 2013 containing 20,221 protein entries, static modification of +57.021 for C residues, variable modifications of +15.995 and +42.011 for M and K residues, respectively, parent ion tolerance of +/−10 ppm, fragment ion tolerance of 0.6 Da, monoisotopic masses, and trypsin cleavage (max 2 missed cleavages). Searches used a reversed sequence decoy strategy to control peptide false discovery [5] and identifications were validated by Percolator software [6]. Only peptides with *q*-values <0.01 were accepted, corresponding to a false discovery rate of below 1%.

#### 1.3.4 Additional analyses of identified acetyl-lysine sites and modified proteins

An assessment of acetyl-lysine protein functional network connectivity and associated gene ontology (GO) biological processes was performed using STRING v9.1 [7] analysis of unique Ensemble protein identifiers of all identified lysine acetylated platelet proteins at a ≥0.7 confidence score. To assess candidate lysine acetyltransferases involved platelet protein acetyl-lysine modification, all identified acetyl-lysine peptide sequences were put into the Acetylation Set Enrichment-Based (ASEB) prediction tool (http://bioinfo.bjmu.edu.cn/huac/predict_p/) and assigned *p*-values predicting modification by p300/CBP and GCN5/PCAF lysine acetyltransferases [8]. To assess the relative abundance of platelet proteins found to contain acK modifications, all platelet proteins with identified acK sites were assigned an abundance rank as previously determined [9].

#### 1.3.5 Data deposition

The mass spectrometry proteomics data have been deposited to the ProteomeXchange Consortium [2] via the PRIDE partner repository with the dataset identifier PXD002332 accessible at http://www.ebi.ac.uk/pride/archive/projects/PXD002332
